# Development of a Plantar Load Estimation Algorithm for Evaluation of Forefoot Load of Diabetic Patients during Daily Walks Using a Foot Motion Sensor

**DOI:** 10.1155/2017/5350616

**Published:** 2017-08-03

**Authors:** Ayano Watanabe, Hiroshi Noguchi, Makoto Oe, Hiromi Sanada, Taketoshi Mori

**Affiliations:** ^1^Department of Gerontology, Graduate School of Medicine, The University of Tokyo, 7-3-1 Hongo, Bunkyo-ku, Tokyo, Japan; ^2^Department of Life Support Technology (Molten), Graduate School of Medicine, The University of Tokyo, 7-3-1 Hongo, Bunkyo-ku, Tokyo, Japan; ^3^Department of Advanced Nursing Technology, Graduate School of Medicine, The University of Tokyo, 7-3-1 Hongo, Bunkyo-ku, Tokyo, Japan

## Abstract

Forefoot load (FL) contributes to callus formation, which is one of the pathways to diabetic foot ulcers (DFU). In this study, we hypothesized that excessive FL, which cannot be detected by plantar load measurements within laboratory settings, occurs in daily walks. To demonstrate this, we created a FL estimation algorithm using foot motion data. Acceleration and angular velocity data were obtained from a motion sensor attached to each shoe of the subjects. The accuracy of the estimated FL was validated by correlation with the FL measured by force sensors on the metatarsal heads, which was assessed using the Pearson correlation coefficient. The mean of correlation coefficients of all the subjects was 0.63 at a level corridor, while it showed an intersubject difference at a slope and stairs. We conducted daily walk measurements in two diabetic patients, and additionally, we verified the safety of daily walk measurement using a wearable motion sensor attached to each shoe. We found that excessive FL occurred during their daily walks for approximately three hours in total, when any adverse event was not observed. This study indicated that FL evaluation method using wearable motion sensors was one of the promising ways to prevent DFUs.

## 1. Introduction

Diabetic foot ulcer (DFU) is one of the serious and prevalent complications of diabetes, and they are defined as cutaneous erosions characterized by a loss of epithelium that extends into or through the dermis to deeper tissues [1]. Diabetes is known to delay wound healing, and 85% of all amputations are the result of a nonhealing DFU [2, 3]. Through many clinical cases, callus has been recognized as a pathway to DFU because tissue damage is caused under the hyperkeratotic plantar epidermis [4]. A previous study reported that 56.3% of all of DFUs were located beneath metatarsal heads (MTHs) [5]. Hyperkeratosis is caused by excessive mechanical loading, which is more likely on bony prominences of the forefoot such as MTHs [6]. Thus, the forefoot is at particular risk of developing DFUs.

There have been few studies investigating plantar load in the daily lives of patients with diabetes, while previous measurements of plantar load during walking have been tried as a means of assessing the at-risk foot and to prevent ulceration [7, 8]. Also, some studies have carried out comparison of plantar load in diabetic patients and healthy control subjects [9, 10]. However, these measurements have been applied generally within laboratory and outpatient settings that specialize in DFU. Furthermore, plantar load measurement is typically limited to walking on a short, level walkway, despite the fact that some clinical cases have indicated development of DFUs might be related to the excessive load occurring in daily walks. For example, diabetic patients who are still working walk more often in their daily lives than the elderly, so their feet are more exposed to the risk of DFUs caused by calluses [11]. Also, there are cases in which custom-made shoes for pressure relief sometimes fail to improve the callus of patients with diabetes who walked a lot routinely [12]. These cases indicate the necessity of plantar load evaluation in the actual daily life of patients such as locations where they walk or their activity. There is thus the possibility that greater load which was not observed in the above settings happens in the patients' daily life environment.

As it stands now, it is challenging technically to establish methods to measure plantar force directly over a long time in daily living. In laboratory settings, two types of plantar force measurement systems are mainly used: platform systems and in-shoe systems [13]. Platform systems are composed of a flat, rigid array of pressure-sensing elements arranged in a matrix configuration and embedded in the floor. Therefore, the use of platform systems is generally restricted to laboratories. In-shoe force measurement systems are flexible and inserted in the shoe. The in-shoe systems are used in various studies of gait but several cables connecting force sensors and a data logger disturb activities in daily living. In addition, soft force sensors are not strong enough to bear continuous load for a long time, whereas rigid force sensors have risk to cause damage on the plantar in long-time measurement.

The human gait cycle can be divided into a stance phase and a swing phase. The stance phase is defined as the duration when the foot is on the ground, which can be subdivided into three phases: heel-strike, midstance, and push-off. Generally, the forefoot load (FL) increases substantially from the beginning of the midstance to the end of the push-off. In other words, midstance and push-off are the period when the forefoot is in contact with the ground.

In order to determine the FL, the midstance and push-off need to be identified. However, calculation for the center of the plantar load is required to recognize the phases using the trajectory of the force center, which is not easy [14]. The plantar load can be considered as equal to the sum of ground reaction forces (GRFs) which are acting between the foot and ground during stance phase. The GRFs can be computed by means of Newtonian mechanics. Hence, in this study, we adopted a different approach that identifies the midstance and push-off phases and then calculates the force localized to the foot, using inertial sensors which provide accelerations and angular velocities that are composition elements of Newtonian mechanics.

As reported in previous papers, many methods to estimate GRFs have been proposed other than Newtonian mechanics. For instance, a regression model was developed to predict peak plantar load from an acceleration-based activity monitor [15]. While the developed model predicted peak plantar load well, it required a dummy variable of the range of locomotion speed which was determined using electronic timing gates. Therefore, this model is workable only under laboratory settings. Also, GRF estimation studies using neural network models have increased recently but they require carefully chosen input variables and many training data for the estimation model to reduce errors in the estimation [16, 17]. The Newton mechanics has been known to be able to calculate GRFs accurately in the most of the stance phase without any complicated process [18]. Yamazaki developed a method that calculates GRFs by solving the force equilibrium equations for each of the body segments of a mechanical model [19]. In his study, changes in body segment orientation and posture during walking were obtained by the optical motion capture system. Typically, the system uses cameras to obtain serial images of motions, which is not practical for daily use. However, it may be possible to alternate the optical motion capture system with the wearable motion sensor systems.

Wearable inertial motion sensors are composed of an acceleration sensor and a gyro sensor, and they have already been popular in biomedical applications, that is, measurement of physical activity in daily living. The wearable motion sensors are small enough to be attached anywhere and constrain or affect their user in any way, so they can be easily deployed in daily use [20, 21].

The purpose of this study was to evaluate the FL in daily walks of diabetic patients using a wearable motion sensor attached to each foot, so as to compare the FL between daily life environment and laboratory setting. In this paper, we first describe the process of FL estimation. Next, we validate the estimated FL by comparing it with FL measured by force sensors on the forefoot, which was assessed using the Pearson correlation coefficient. The estimated FL calculated using seven motion sensors of all the lower body segments, as well as only using a motion sensor of each foot, is validated here. Finally, we evaluate the FL of two diabetic patients estimated from foot motion data so as to determine the differences in the FL between their actual daily environment and the laboratory setting such as a short level corridor.

## 2. Methods

This study is composed of two main experiments. The first experiment is to validate estimated FL using correlation with FL measured by force sensors among healthy subjects. The walking measurements for the validation of the estimation algorithm were carried out in the following places: a level corridor, stairs, and a slope. The second experiment is to compare the FL between their actual daily environment and laboratory setting.

Written consent was obtained prior to the study, and all procedures were approved by the Research Ethics Committee of the Graduate School of Medicine, the University of Tokyo (number 11343).

### 2.1. Development of the Algorithm for Estimation of Forefoot Plantar Load

#### 2.1.1. Subjects

Ten healthy subjects (4 males and 6 females; age: 32 ± 9 yr; weight: 61 ± 16 kg) without walking disorders participated in the experiment.

#### 2.1.2. Instrumentation

**Figure 1:**
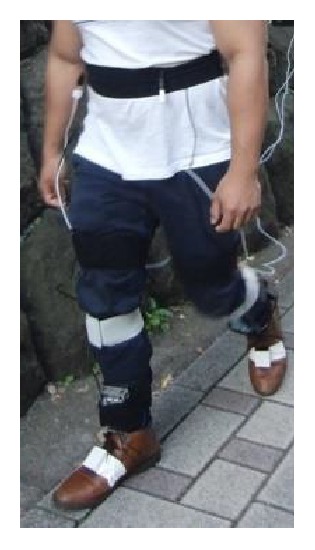
Walking measurement for validation of the estimation algorithm. A motion sensor was attached to each of the lower body segment; in addition, 4 force sensors were attached to 1st and 2nd MTH (2 sensors each).

**Figure 2:**
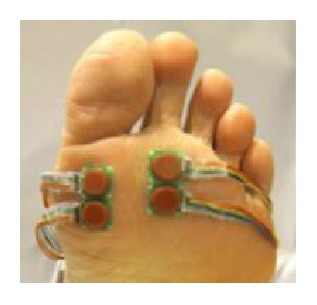
Force sensors on the 1st and 2nd MTH used for validation of the estimation algorithm.

Seven inertial motion sensors containing a 3-axis acceleration sensor and a 3-axis gyro sensor (Logical Product Corporation, Fukuoka, Japan) were attached to several locations: sacrum, left and right thigh, left and right shank, and left and right foot (Figure 1). The motion sensors were secured to each body segment by Velcro straps so as to measure motion in the sagittal plane. Acceleration and angular velocity were recorded at a sampling rate of 100 Hz and low pass filtered using a fourth order, zero-lag critically damped filter with a cut-off frequency of 20 Hz [22]. It should be noted that synchronization with motion data of the seven body segments did not always work correctly. That was why one of the seven motion sensors tended not to collect data at a determined frequency. It was difficult to interpolate when much data were missed consecutively. Four triaxial force sensors (Touchence Inc., Tokyo, Japan) were attached to the 1st and 2nd metatarsal head (MTH) as shown in Figure 2 [23]. The force sensors are able to measure up to 40 N with a sampling rate of 100 Hz.

#### 2.1.3. Protocol

Subjects walked at a self-selected forward speed in the following settings: a 15 m long corridor, stairs consisted of 10 steps which had a 300 mm tread and 180 mm height, and a 15 m long slope with an inclination of 4°. Ascending and descending motion were both performed on the stairs and slope. They walked wearing the shoes they use routinely for daily activities.

#### 2.1.4. Estimation of Forefoot Plantar Load

**Figure 3:**
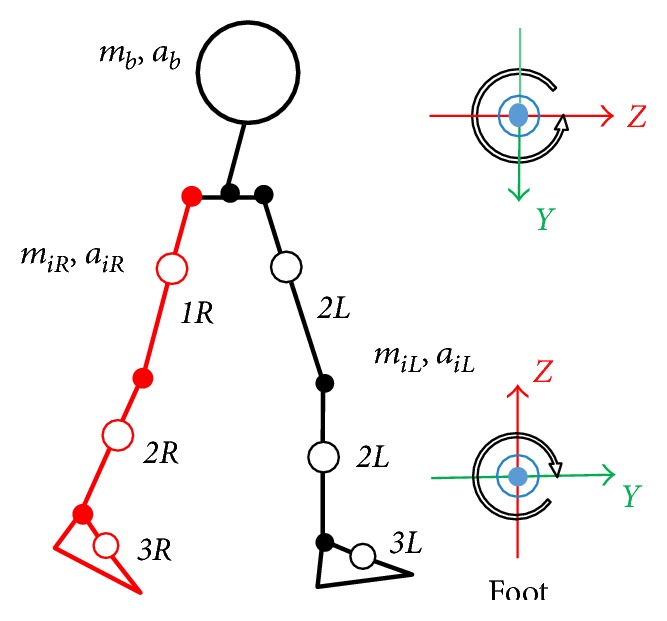
Rigid link model employing the present study and local coordinate system.

In this section, first, we calculate vertical GRF based on Newton's equation of motion; then, we discriminate the midstance and push-off to estimate the FL. A seven-rigid-link model constructed by Yamazaki [19] was used in this study (Figure 3()). All motions were assumed to take place in the sagittal plane. The plantar load *N* was computed by means of Newton's equation of motion, which states that the sum of all external forces balances the sum of the mass-acceleration products of all individual body segments as follows:

(1) $$\begin{array}{c} N=m_{b}\left(a_{b}+g\right)+\overset{3}{\underset{i=1}{\sum }}m_{i}\left(a_{iR}+a_{iL}+2g\right). \end{array}$$

Here, *m*_*b*_ was the mass of the upper body, *a*_*b*_ was the vertical acceleration of the upper body, *m*_*i*_ was the mass of the *i*th leg segment, *a*_*iR*_ was the vertical acceleration of the *i*th right leg segment, *a*_*iL*_ was the vertical acceleration of the *i*th left leg segment, and *g* was the gravitational constant (9.8 ms^−2^). The mass of each segment was calculated from the body weight based on the anthropometric study data [24]. Vertical acceleration was computed from the acceleration and integrated angular velocity (*θ*). The angular velocity integration was commenced from tilt angles of each body segment which were computed using static acceleration. Vertical acceleration of the foot could be expressed as

(2) $$\begin{array}{c} a_{3}=a_{3z}\cos\theta_{3}-a_{3y}\sin\theta_{3}, \end{array}$$

where *a*_3*y*_ denoted the foot acceleration of the *y-*axis and *a*_3*z*_ denoted the foot acceleration of the *z-*axis. Vertical acceleration of the thigh could be written as

(3) $$\begin{array}{c} a_{1}=a_{1z}\sin\theta_{1}-a_{1y}\cos\theta_{1}, \end{array}$$

where *a*_1*y*_ was the thigh acceleration of the *y-*axis and *a*_1*z*_ was the thigh acceleration of the *z-*axis. Vertical acceleration of the shank could be expressed as

(4) $$\begin{array}{c} a_{2}=a_{2z}\sin\theta_{2}-a_{2y}\cos\theta_{2}, \end{array}$$

where *a*_2*y*_ was the shank acceleration of the *y-*axis and *a*_2*z*_ was the shank acceleration of the *z-*axis.

**Figure 4:**
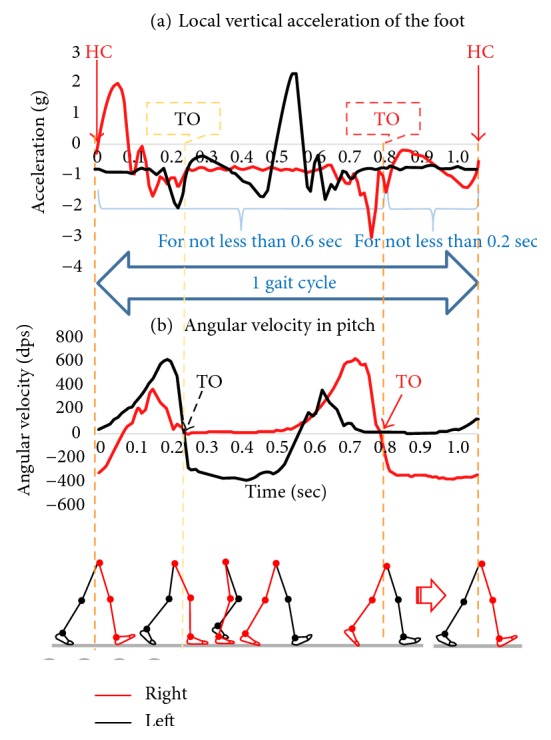
Algorithm discriminating IC and TO by foot acceleration and angular velocity.

To detect stance phase based on foot motion data, heel contact (HC) is defined as

(5) $$\begin{array}{c} a_{3Zt}\cdot a_{3z\left(t+1\right)}\leq 0, \end{array}$$

and the period from TO of the contralateral foot to HC of the ipsilateral foot is ≥0.2 sec, where *a*_*Zt*_ denoted the foot acceleration of the *z-*axis at *t* sec. Toe off (TO) is defined as

(6) $$\begin{array}{c} r_{3xt}\cdot r_{3x\left(t+1\right)}\leq 0, \end{array}$$

and the period from HC to TO of the ipsilateral foot is ≥0.6 sec, where *r*_*xi*_ denoted the angular velocity in pitch at *t* sec (Figure 4). The time involving stance phase and swing phase is determined based on the percentage of time involved in the stance phase and swing phase [25]. Also, the minimum time spent on the phases, which was determined empirically, was taken into account.

In order to recognize a period when the forefoot is contact with the ground in the stance phase, a period from the beginning of the midstance to TO needs to be identified. In this study, the beginning of midstance was defined as TO of the ipsilateral foot.

The pattern of the forefoot force while walking down the stairs was diverse among the subjects because the foot location touching a tread at the HC varied among them. Therefore, it is difficult to apply a consistent algorithm to all of the walking on a level corridor, stairs, and a slope. Hence, the descending stairs were excluded from the adaptation of the estimation algorithm.

#### 2.1.5. Validation of Forefoot Load Estimation Algorithm

To validate the estimation algorithm, the estimated FL was compared with the FL measured by force sensors. In this study, the estimation algorithm was validated using correlation between the estimated FL and the measured FL because we aimed to detect relative excessive load in intrasubject. The correlation was assessed by the Pearson correlation coefficient. First, we computed correlation during midstance and push-off of each step, which was equal to approximately 0.6 sec (60 samples). Then, we calculated the mean and standard deviation (SD) of the Pearson correlation coefficient over 30 steps (15 steps of each foot) excluding the 1st step and last. In the case of the stair walking, the mean and SD of the Pearson correlation coefficient over 9 steps (4 steps of one foot and 5 steps of the other) excluding the last step were assessed. The Pearson correlation coefficient was categorized (in absolute value) as *p* ≤ 0.35:weak, 0.35<*p* ≤ 0.65:moderate, 0.67<*p* ≤ 0.9:strong, 0.9<*p*:excellent [26]. One of the four force data representing the maximum value during midstance and push-off was used as the reference data of estimated FL.

Finally, 18 of the 50 trials, which were measured without the errors in sampling, were included in the correlation analysis.

#### 2.1.6. Applicability of the Algorithm

The estimated FL and FL measured by force sensors on the MTHs demonstrated from moderate to strong correlation during walking on a level corridor (Table 1(a)). The magnitude of correlation remained consistent even when the FL was estimated only using foot motion data. The mean of correlation coefficient of all the subject was 0.63.

In the other places, the correlation between the estimated FL and FL measured by force sensors on the MTHs showed an intersubject difference (Tables 1(b), 1(c), and 1(d)).

Finally, we could demonstrate that the estimation algorithm was applicable to walks on the level ground. However, the estimation algorithm was not adequate to use walks on stairs or slopes.

### 2.2. Plantar Load Measurement in Daily Living of Patients with Diabetes

#### 2.2.1. Subjects

Two diabetic patients participated in the study and their characteristics are as shown in Table 2. This time, patients under 60 years old, who often go out and walk routinely, were included in this study. In addition, from the aspect of safety, patients with neuropathies or calluses were excluded.

#### 2.2.2. Instrumentation

**Figure 5:**
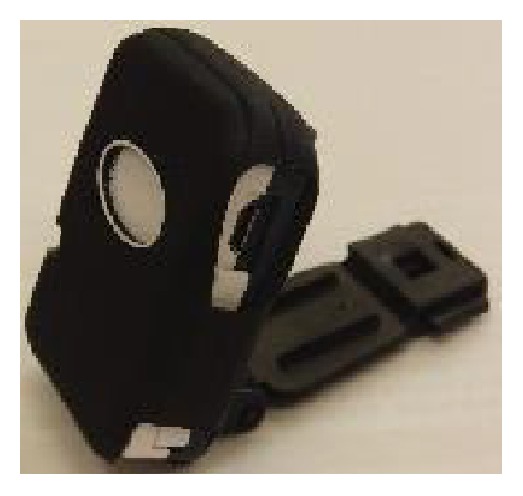
A motion sensor used for daily walk measurement.

**Figure 6:**
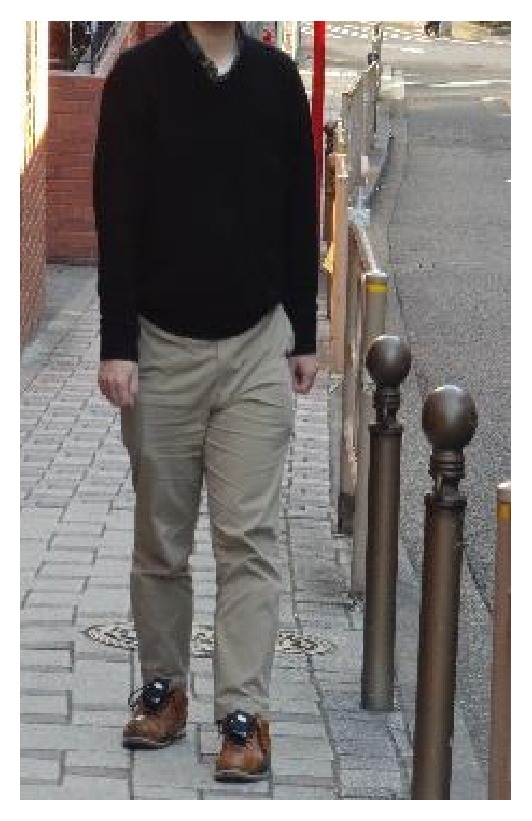
Walking measurement for identification of the places where the excessive FL occurs in daily walks. A motion sensor was attached to each shoe.

A motion sensor containing a 3-axis acceleration sensor and a 3-axis gyro sensor (ATR-Promotions Inc., Soraku, Japan) were attached to each shoe with strap (Figures 5 and 6). Acceleration and angular velocity were recorded at a sampling rate of 100 Hz.

#### 2.2.3. Protocol

*(1) Walking Measurement in Laboratory Setting*. Subjects walked at a self-selected forward speed on a 15 m long corridor twice, wearing the shoes they use routinely for daily activities.

Thirty steps during walking corridors were evaluated, which did not include the first and last steps.

*(2) Walking Measurement in Daily Life Environment*. After the subjects walked on the corridors, they were asked to record their foot motion while they walked wearing the shoes in their daily activity area. Also, they were asked to take notes where they were walking and whether they used vehicle when they traveled from place to place.

In daily walk data, consecutive steps for not less than 30 seconds were analyzed, because the period was assumed to be longer than the common walking measurement time in laboratory settings.

*(3) Definition of the Excessive Forefoot Load*. A walking trial on a 15 m long corridor was performed twice to define the “excessive load” of each patient from either one of the trials. Estimated FL points deviating more than 2 standard deviations from the average maximum FL of 15 steps were defined as excessive FL. The frequency of the excessive FL during walking was compared between the two settings: a corridor and daily life environment.

## 3. Results

Excessive FL of DM01 and DM02 were defined as over 10.0 kgf and 3.2 kgf each (Table 3).

The FL which exceeded 10.0 kgf was not observed in his 30 steps when DM01 was walking on a level corridor. DM02 also did not have the FL over 3.2 kgf in her 30 steps on the level corridor.

In DM01, motion data for approximately 3.0 hours (10,800 seconds) was recorded. This included going to the train station from home by bus, going to have lunch by train, and moving to another station to get off the train. The total time spent on consecutive steps for not less than 30 seconds within the said hours was 731.2 seconds. In addition, the average time was 1.3 seconds for each gait cycle. The sum of all of the consecutive steps over 30 sec was 2126 steps, in which excessive FL occurred 151 times. Excessive FL happened most frequently during walking from home to the bus stop, and 48 of the 238 steps exceeded 10 kgf. A gait cycle took about 1.07 sec then. The maximum excessive FL of the 2126 steps was 14.1 kgf, which occurred for 18 seconds after he got off a train, when each gait cycle took approximately 1.2 seconds.

In DM02, motion data for approximately 3.2 hours (12,000 seconds) was recorded. This included walking to the station from the restaurant, going shopping to downtown by train, and going home. Total time spent on consecutive steps for not less than 30 seconds within the said hours was 2336.0 seconds. In addition, the average time was 1.2 seconds for each step cycle. It was determined that 4030 steps in total during the consecutive steps period and 762 steps within the 4030 steps demonstrated excessive FL. Excessive FL most frequently occurred while walking back home from downtown, during which time 186 of the 372 steps were counted as demonstrating excessive load. At this time, each gait cycle was approximately 1.04 seconds. The maximum excessive FL was 6.3 kgf which occurred while DM02 was walking on a concrete sidewalk, when one gait cycle took about 1.2 sec.

After all, the excessive FL was not observed in both patients in the laboratory setting. By contrast, both of them had excessive FL in their daily life environment. The excessive FL occurred at a rate of 1 time per about 14 steps in daily walks of DM01. Also, the excessive FL happened at a rate of 1 time per about 5 steps in daily walks of DM02.

## 4. Discussion

This is the first study to investigate the forefoot load of diabetic patients in a daily life environment using foot motion data.

Daily walk measurement using wearable motion sensors appears feasible and safely induces no adverse events in patients with diabetes. The smallness and lightness of the inertial wearable motion sensors were considered to allow daily measurement of daily walks in diabetic patients.

Daily walks of two diabetic patients were measured and consecutive steps not less than 30 seconds were analyzed. The sum of all of the consecutive steps that were timed did not occupy much of the recorded time of DM01 because he was mainly traveling by buses and trains. DM01 had excessive FL at a rate of 1 per 5 steps on his way from his home to the bus stop. The daily walking data of DM02 included the time strolling around for shopping downtown, and consecutive steps that took less than 30 seconds were often observed. However, it took approximately 2 seconds for one gait cycle, which was relatively slow. Therefore, excessive FL can be assumed to have seldom occurred during walking when the purpose of the consecutive steps was not to move around [27]. DM02 had excessive FL most frequently when going home from downtown and the rate was 1 of 2 steps. It was observed that there was no excessive load when 30 steps were counted as having been walked on a corridor. Thus, a patient whose forefoot tends to receive excessive load could not be found unless the walking measurement is taken in a daily life environment. The situations in which excessive FL occurred may be different from a laboratory setting in terms of the walking speed or properties of the road surface in their daily life environment [28]. Therefore, these differences may suggest the occurrence of excessive FL.

The algorithm was able to estimate the forefoot load during walking on a level corridor with more than moderate accuracy. However, the estimation accuracy was not consistent among the subjects in stair walking and slope walking. If the peak of the FL comes early in the stance phase, correlation between FL and estimated FL was attenuated. Generally, the pattern of the FL and its magnitude would be different depending on places to be walked [29]. For instance, more GRF is applied on the forefoot because impact by a falling body when walking down the slope is greater than when walking on a level floor. These properties may result in attenuating the accuracy of FL estimation. The changes of posture when the center of body mass is raised can be captured accurately by the acceleration of the trunk. In addition, a previous study showed that the pattern of trunk acceleration in the stance phase was similar to GRF [30]. The accuracy of the estimation algorithm would be improved by a combination of trunk and feet acceleration.

Several limitations of this study need to be acknowledged. First, the estimation algorithm proposed here is not applicable when walking down stairs. Foot location at HC is different for each person; some people initiate their landing from the toe and others land from the heel. Hence, algorithm for the different types of landing needs to be established. Second, forefoot load in horizontal component was not estimated despite the fact that shear stress is associated with callus formation [31]. Future studies need to be conducted that consider the forefoot load of both components. Finally, FL under each foot cannot be determined when both feet are in contact with the ground in this study. However, FL of one foot cannot exceed the sum of each FL; thus, the developed algorithm does not underestimate the FL.

We should conduct future research that estimates the daily forefoot load of diabetic patients with/without frequent excessive FL and investigate the association between the frequent excessive FL and calluses. Such a future study would provide insights into the screening of patients at risk for DFU.

## 5. Conclusion

This study created an algorithm to estimate the forefoot load and revealed that excessive load not observed in laboratory settings using level floors did in fact occur during daily walks. In addition, this study demonstrated the feasibility of long-time FL measurement in patients' daily environment. Our next interest is to demonstrate whether excessive load is related to callus formation.

## Figures and Tables

**Table:** (a) Walking on a level corridor

All the motion data of the lower body segments			Only each foot motion data		
ID	Mean ± SD of the Pearson correlation coefficient		ID	Mean ± SD of the Pearson correlation coefficient	
	Left	Right		Left	Right
3	0.68 ± 0.08	0.58 ± 0.10	3	0.72 ± 0.06	0.71 ± 0.05
5	0.07 ± 0.03	0.69 ± 0.10	5	0.69 ± 0.04	0.71 ± 0.04
6	0.62 ± 0.09	0.71 ± 0.05	6	0.67 ± 0.04	0.55 ± 0.06
8	0.68 ± 0.08	0.83 ± 0.04	8	0.61 ± 0.04	0.58 ± 0.09
10	0.07 ± 0.17	0.47 ± 0.14	10	0.57 ± 0.08	0.48 ± 0.10

**Table:** (b) Walking up stairs

All the motion data of the lower body segments			Only each foot motion data		
ID	Mean ± SD of the Pearson correlation coefficient		ID	Mean ± SD of the Pearson correlation coefficient	
	Left	Right		Left	Right
2	0.72 ± 0.06	0.66 ± 0.18	2	0.10 ± 0.23	−0.21 ± 0.04
4	0.65 ± 0.11	0.43 ± 0.16	4	0.39 ± 0.30	0.10 ± 0.56
6	0.67 ± 0.05	0.74 ± 0.04	6	0.67 ± 0.05	0.74 ± 0.04
8	0.74 ± 0.07	0.44 ± 0.07	8	0.68 ± 0.09	0.64 ± 0.10
10	0.38 ± 0.15	0.42 ± 0.14	10	0.05 ± 0.19	0.44 ± 0.16

**Table:** (c) Walking up a slope

All the motion data of the lower body segments			Only each foot motion data		
ID	Mean ± SD of the Pearson correlation coefficient		ID	Mean ± SD of the Pearson correlation coefficient	
	Left	Right		Left	Right
3	0.42 ± 0.28	0.27 ± 0.39	3	0.72 ± 0.03	0.57 ± 0.07
4	0.07 ± 0.15	0.55 ± 0.13	4	0.43 ± 0.13	0.54 ± 0.19
5	0 ± 0.13	0.23 ± 0.09	5	0.57 ± 0.09	0.57 ± 0.12
8	0.54 ± 0.35	0.60 ± 0.34	8	0.42 ± 0.21	0.23 ± 0.36
10	0.30 ± 0.19	0.11 ± 0.31	10	0.29 ± 0.25	0.26 ± 0.20

**Table:** (d) Walking down a slope

All the motion data of the lower body segments			Only each foot motion data		
ID	Mean ± SD of the Pearson correlation coefficient		ID	Mean ± SD of the Pearson correlation coefficient	
	Left	Right		Left	Right
5	0.48 ± 0.11	0.67 ± 0.07	5	0.72 ± 0.03	0.75 ± 0.09
8	0.76 ± 0.08	0.74 ± 0.08	8	0.38 ± 0.25	0.32 ± 0.23
10	−0.17 ± 0.59	−0.07 ± 0.46	10	0.17 ± 0.35	0 ± 0.30

**Table 2:** Characteristics of each patient.

	DM01	DM02
Age (yrs)	43	38
Sex	Male	Female
Diabetes type	2	1
Diabetes duration (yrs)	3	8
HbA1c (%)	8.3	7.2
Height (m)	1.73	1.57
Weight (kg)	105	55
Neuropathy (+/−)	—	—
Present other diseases	Hypertension Hyperlipidemia	—
Occupation	System engineer	Nutritionist

**Table 3:** Excessive FL of each patient.

	DM01	DM02
Mass of the foot (kg)	1.2	0.6
Average estimated FL (kgf)	7.0	1.5
SD of estimated FL (kgf)	1.8	0.84
Excessive FL (kgf)	≧10.0 kgf	≧3.2 kgf
